# Biological Features of Nanoparticles: Protein Corona Formation and Interaction with the Immune System

**DOI:** 10.3390/pharmaceutics14122605

**Published:** 2022-11-26

**Authors:** Sonia Panico, Sara Capolla, Sara Bozzer, Giuseppe Toffoli, Michele Dal Bo, Paolo Macor

**Affiliations:** 1 Department of Life Sciences, University of Trieste, 34127 Trieste, Italy; 2 Experimental and Clinical Pharmacology Unit, Centro di Riferimento Oncologico di Aviano (CRO), Istituto di Ricovero e Cura a Carattere Scientifico (IRCCS), 33081 Aviano, Italy

**Keywords:** nanoparticles, protein corona, immune system

## Abstract

Nanoparticles (NPs) are versatile candidates for nanomedical applications due to their unique physicochemical properties. However, their clinical applicability is hindered by their undesirable recognition by the immune system and the consequent immunotoxicity, as well as their rapid clearance in vivo. After injection, NPs are usually covered with layers of proteins, called protein coronas (PCs), which alter their identity, biodistribution, half-life, and efficacy. Therefore, the characterization of the PC is for in predicting the fate of NPs in vivo. The aim of this review was to summarize the state of the art regarding the intrinsic factors closely related to the NP structure, and extrinsic factors that govern PC formation in vitro. In addition, well-known opsonins, including complement, immunoglobulins, fibrinogen, and dysopsonins, such as histidine-rich glycoprotein, apolipoproteins, and albumin, are described in relation to their role in NP detection by immune cells. Particular emphasis is placed on their role in mediating the interaction of NPs with innate and adaptive immune cells. Finally, strategies to reduce PC formation are discussed in detail.

## 1. Introduction

Nanomedicine is “a branch of medicine that applies the knowledge and tools of nanotechnology to the prevention and treatment of diseases” [1]. In recent years, novel nanotechnologies have attracted a lot of interest due to their potential applications in the medical field. This is consistent with recent reports published by the Business Communications Company, which estimated the global market for nanomedicine to be approximately USD 53 billion in 2009. This is expected to grow to USD 293.1 billion by 2022 [2].

Nanomedicine is based on the use of structures that range between 1 and 100 nm in diameter, which are called “nanoparticles” (NPs). The first nanosized drug approved by the Food and Drug Administration (FDA) was Doxil^®^, i.e., doxorubicin encapsulated inside lipid NPs, which is used to treat multiple myeloma, benign ovarian cancer and soft-tissue sarcoma. The success of nanomedicine is related to the many strengths of NPs, which lie in their tunable biochemical, electronic, magnetic, and optical properties, making them suitable for targeting specific organs or tissues. This specificity strongly reduces the systemic toxicity of conventional therapies while increasing drug concentrations in the pathological microenvironment, which improves drug efficacy [3,4]. However, even when the therapeutic efficacy of a nanomedicine has been demonstrated via preclinical and clinical trials, many drugs have been discontinued after years on the market. Indeed, a success rate of only 11.2% has been estimated [5]. The reason for this is the poor clinical translation of nanomaterials due to the gap between preclinical and clinical studies, which is mainly due to the differences between animal models and humans in terms of biodistribution, pharmacokinetics, and even side effects after in vivo administration. In addition, compared to conventional drugs, nanomedicines are characterized by greater complexity in predicting the variability of response rates and therapeutic efficacy. In terms of toxicity studies, encapsulation of drugs in NPs dramatically alters the biodistribution and organ exposure, potentially leading to toxic effects and/or reduced efficacy. Another aspect that concerns the safety is the variation in immunological responses after administration; an example is the hypersensitivity reactions that occur in a small percentage of patients, but still represent a limit for the application of nanomedicine [6]. In general, the cytotoxicity and side effects of each NP candidate vary from case to case, further complicating clinical translation. Another key factor is the interaction between the NPs and the biological environment of the systemic circulation, which is mainly characterized by the emergence of a dynamic interplay between the circulating proteins and the NPs themselves. This interaction leads to the formation of a protein layer surrounding the NPs, the so-called protein corona (PC) [7], which strongly impacts NPs’ biodistribution, immunogenicity [8,9], and physical properties, including physical destabilization and agglomeration [10]. For these reasons, the PC has long been considered a biological barrier that must be overcome for effective NP-based therapy.

Several strategies have been proposed to overcome the formation of the PC, thus improving the stability of NPs and extending their circulation time. This has been achieved by using synthetic or biological materials for biomimetic approaches, commonly defined as “stealth-inducing” or “fouling-inhibiting” materials that reduce the interaction of NPs with immune system components and cells [11,12]. On the other hand, controlling the formation of PC could be used to modulate the immune response to NPs and ultimately improve the efficacy of NP-based therapy by exploiting interactions with immune system players rather than simply avoiding them entirely [13,14,15].

This review focuses on the interaction between NPs and the immune system through mechanisms mediated by PC formation. First, we define the general characteristics of the PC and the main intrinsic properties of NPs, as well as the extrinsic factors that influence PC formation. The role of the PC in the interaction between NPs and cells of the immune system is highlighted, with particular attention paid to cells of the innate and adaptive immune systems. Finally, the main strategies for controlling the evasion of the immune system by NPs are described.

## 2. The Protein Corona

### 2.1. General Features

**Figure 1:**
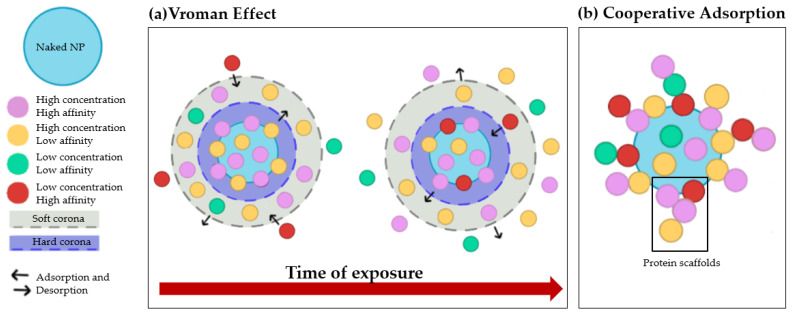
Overview of the phenomena that affect protein adsorption on nanoparticles. (**a**) The Vroman effect: overtime proteins with higher affinity displace proteins with lower affinity. (**b**) Cooperative adsorption: already bound proteins act as scaffold for other proteins on the NPs’ surface.

The PC is a dynamic, multilayered structure formed by the interaction of NPs with highly concentrated proteins in biological fluids, especially blood. It is now known that the PC alters identity, size, and surface charge of NPs [12,15], which in turn has implications for NPs’ interactions with cell membranes and their internalization pathways, cytotoxicity, targeting ability, and clearance in vivo [16,17]. Consequently, further studies of the PC are essential for a more accurate prediction of the effects of NPs after injection, especially when used as drug delivery systems (DDSs) [12,15]. The first level of categorization of proteins bound to NPs is represented by the distinction between “hard” and “soft” PCs, designations that describe the dynamic nature of this phenomenon. Specifically, the hard PC consists of high-affinity proteins that are rapidly and directly adsorbed onto the NPs’ surface and remain stable over time. On the other hand, proteins that interact only weakly with the already bound hard corona form the so-called “soft PC”. The main difference between hard and soft corona lies in the native conformation of adsorbed proteins. In soft corona, proteins maintain their native conformation, while in hard corona, they undergo changes in their structure [18,19]. These conformational changes are of concern for two reasons: (i) a possible loss of protein activity, which is highly dependent on the tertiary/quaternary structure, and (ii) the formation of new epitopes that could trigger an immune response. Therefore, it is crucial to investigate not only the composition, but also the conformation of proteins adsorbed by NPs. These assessments are complicated by the constant adsorption and desorption of proteins, a process that depends on two phenomena: the Vroman effect and cooperative adsorption. The Vroman effect consists of competitive adsorption and desorption of proteins on the surface of NP. The most abundant proteins are first adsorbed to the NPs and then replaced by those with higher affinity, regardless of their concentration [16,20] (Figure 1a). On the other hand, cooperative adsorption highlights the ability of already bound proteins to act as scaffolds for other proteins; indeed, it appears that some proteins may adsorb more strongly to the corona when other proteins are present (Figure 1b).

### 2.2. Intrinsic NPs Properties That Affect the PC Formation

The physicochemical properties of NPs, such as size, surface curvature, shape, charge, hydrophobicity, and surface structure, can influence the PC’s behavior.

The effect of the size of NPs on the PC formation is well documented in the literature, but with some discrepancies. Hu et al. investigated the effects of size on the formation of PC after incubating iron oxide (Fe_3_O_4_) NPs of increasing size (30, 200, 400 nm) with human plasma (HP). The number of bound proteins was almost the same (117, 133 and 100 proteins on 30 nm-, 200 nm-, and 400 nm-sized NPs, respectively), but only 20% of the PC was shared among the particles, indicating the influence of particle dimension on the type and abundance of adsorbed proteins. An example of this is human serum albumin (HSA), which was the second most abundant protein adsorbed on 200 nm-Fe_3_O_4_ NPs, but was only the 12th and 27th most abundant protein on the PC of 400 nm- and 30 nm-Fe_3_O_4_ NPs, respectively. This difference must be taken into account, as it may have an impact on the biological fate of the NPs [21]. The demonstrated difference in the PC composition based on the dimension of NPs also applied to 50 nm- and 100 nm-sized carboxyl-modified polystyrene NPs (PS-NPs), which shared only ~50% of the adsorbed proteins. However, these data were in contrast with those obtained from the analysis of 50 nm- and 100 nm-sized neutral PS-NPs. In fact, the PC of these two nanostructures was very similar, with a homology of ~80%. The size of NPs was also shown to affect the adsorption of proteins such as immunoglobulins (Igs) and apolipoproteins (Apos), which mediate or prevent the recognition of NPs by immune cells. Specifically, larger PS-NPs exhibited higher abundance of Igs in PC compared to smaller particles, and the same pattern was visualized for Apo-B100 [22]. The size is also related to the surface area-to-volume ratio, a parameter that is increased in small particles. Consequently, a larger number of proteins can be accommodated by smaller nanostructures than by larger ones when considering a normalized surface area. Based on simple geometric considerations, it is also possible to estimate the maximum number of proteins that can be adsorbed on NPs based on their size. An example is silica NPs (SiO_2_-NPs) with a diameter of 26 nm, which have been shown to adsorb 27 fibrinogens (molecular weight (MW): 340 kDa) or 30 histidine-rich glycoproteins (MW: 70 kDa), or 47 kininogen-1 molecules (MW: 110 kDa) [23].

**Figure 2:**
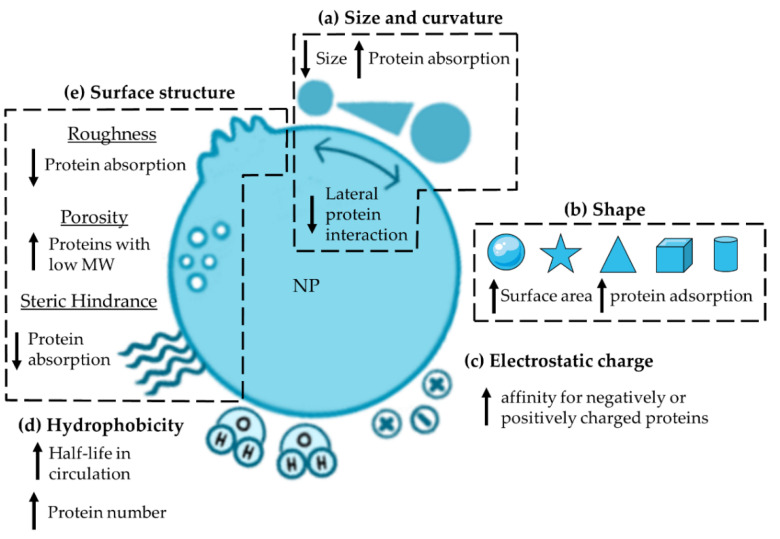
Overview of the intrinsic properties that influence PC formation: (**a**) size and curvature, (**b**) shape, (**c**) electrostatic charge, (**d**) hydrophobicity and (**e**) surface structure. MW: molecular weight; NP: nanoparticle.

Curvature is another intrinsic factor that is closely related to the size of NPs and plays an essential role in the formation and composition of PC. Specifically, smaller NPs (diameter < 10 nm) are characterized by higher surface curvature than larger ones (diameter > 100 nm), which can be considered as flat surfaces [24]. Based on this assumption, smaller NPs are expected to adsorb a larger number of proteins, which is associated with less lateral protein interaction, resulting in higher adsorption of proteins that normally repel each other [12]. In addition, the stronger curvature of small NPs optimizes the packing of proteins on the surface, while a lower curvature is characterized by steric hindrances between neighboring proteins, making the whole surface less accessible. To confirm this, SiO_2_ NPs with larger diameters (10, 30 and 80 nm) were used to show how the lower curvature in each case affects the maximum number of bound proteins, but not the PC composition (Figure 2a) [24].

The influence of shape in the PC formation has been highlighted in several studies using gold (Au) or zinc (Zn) NPs with different morphologies. Based on the concept that spherical (sAuNPs) and branched-shaped AuNPs (bAuNPs) are the two main configurations for biomedical applications, these two structures were compared. In particular, the binding of HSA to sAuNPs and bAuNPs with almost the same dimensions (50 nm and 64 nm of the core, respectively; branches of bAuNPs: 16 nm) was studied. In detail, the differences between the particles lie in the HSA affinity, the type of layer formed, and the orientation. HSA showed three times higher affinity for sAuNPs than to bAuNPs; moreover, it formed a monolayer on sAuNPs, on which it is oriented mainly in one direction, while it filled the gaps between the branches of bAuNPs with a multi-oriented binding pattern. All these features also affected the thickness of the formed PC, which was larger on bAuNPs than on sAuNPs [25]. Further studies comparing Au nanorods (AuNRs) and Au nanostars (AuNSTs) showed a significantly higher number of proteins adsorbed on AuNSTs, probably due to the larger surface area compared to AuNRs [26]. This confirms what was previously shown for sAuNPs and bAuNPs (surface-area-to-volume: sAuNPs > bAuNPs) [25]. The same pattern was confirmed when comparing AuNRs, sAuNPs, AuNSTs, and Au nano-cages (AuNCs) with a size of ~50 nm. In fact, the highest amount of proteins per weight was detected on AuNSTs, as they have a larger surface-to-volume ratio compared to the other particle types. In contrast, AuNCs, which had the smallest surface area and greater curvature compared to sAuNPs, had lower total protein abundances. The shape of the particles also affects the composition of the PC (Figure 2b). Although AuNCs adsorb a lower total amount and diversity of proteins compared with other AuNPs, a greater abundance of albumin and C1q, a component of the classical complement (C) system activation pathway, and a lower amount of vitronectin—a protein that inhibits C activation—were detected. These binding patterns could indicate a possible variation in the cellular uptake of the particles [27].

The electrostatic charge of NPs has been shown to affect the PC composition. Comparison of three different surface chemistries, such as neutral PS-NPs, amine-modified PS-NPs (NH_2_-PS-NPs, positive charge), and carboxyl-modified PS-NPs (COOH-PS-NPs, negative charge) supports this theory. Despite 35–40% common proteins in all three coronas, about 35% of the PC was unique to each particle type. The remaining 25–30% of proteins were shared by NH_2_-PS or COOH-PS-NPs and neutral NPs [22]. Regarding the identity of adsorbed proteins, since most of them are negatively charged at physiological pH, cationic NPs are thought to adsorb a larger number of proteins than anionic particles; however, this is not the rule (Figure 2c). An example is albumin, which carries a negative charge at physiological pH (isoelectric point (IP) below 5.5) [28] and consequently binds preferentially to positively charged NPs. However, positively and negatively charged colloidal AuNPs were shown to adsorb the same number of albumin molecules, refuting this theory [29]. What has been evidenced in the published studies is the higher affinity of some proteins for negatively or positively charged surfaces. This is the case for Igs, which showed a higher affinity for COOH- than NH_2_-AuNPs carrying a negative or positive charge, respectively [29]. The same pattern was observed with differently charged PS-NPs [30] and with synthetic latex NPs prepared ad hoc to address this issue [28]. In other proteins, positively charged PS-NPs have also been shown to adsorb more Apo-F and C components, such as C1r and mannose-binding protein (MBL), than their negatively charged counterpart, affecting their immunotoxicity [30].

Hydrophobicity affects the formation and identity of PC. Indeed, the number of identified proteins was significantly higher with increasing hydrophobicity; hydrophobic nanogels (NGs) bound ~9 × 10^9^ proteins, while the amount of bound proteins on the hydrophilic counterpart was ~2 × 10^9^ (Figure 2d). Specifically regarding the PC composition, hydrophobic NGs adsorbed a smaller amount of Apo-E and albumin than hydrophilic NGs [31]. This pattern was also confirmed for sAuNPs; the introduction of hydroxyl groups on their surface resulted in a significant decrease in the binding of HSA and IgE, while Apo-E was not affected, thus prolonging the half-life of the particles after injection [32]. Moreover, studies on PS-poly(*N*-isopropylacrylamide) (PNIPAM) NPs showed clusterin (Apo-J) enrichment on a hydrophilic surface, while Apo-E and Apo-A1 showed an opposite pattern, as previously reported [33].

Other surface parameters that affect protein adsorptions include roughness, porosity, and steric hindrance (Figure 2e). Piloni et al. investigated the effect of roughness and demonstrated that compared to smooth polymer-grafted NPs, those grafted with patchy polymers decreased protein adsorption [34]. As for porosity, it does not increase protein adsorption but enhances the deposition of proteins with low MW [35]. Steric hindrance can be modified by grafting polymers such as polyethylene glycol (PEG) onto the surface of NPs. In particular, the density and conformation of such polymers have been shown to affect the accessibility of proteins on the surface. In fact, it has been demonstrated that increasing density of PEG significantly reduces protein adsorption and consequently alters the composition of the protein layer [36].

### 2.3. Extrinsic Factors which Affect the PC Formation

In addition to intrinsic factors that are closely related to the composition and properties of NPs, there are also extrinsic factors that influence the formation of PC in vitro. In particular, solutions conditions, dynamics, and biomolecular conditions should be mentioned.

**Figure 3:**
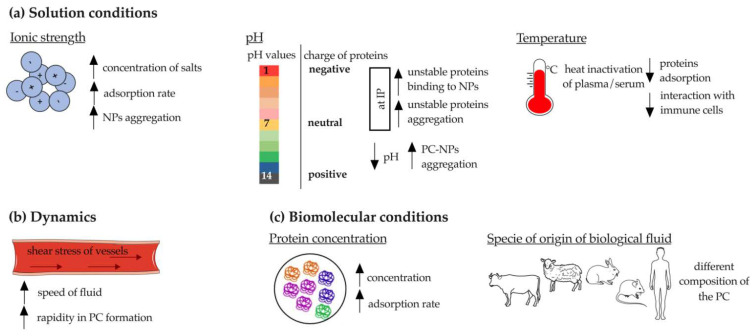
Schematic representation of extrinsic factors affecting the protein corona formation. (**a**) Solution conditions; (**b**) dynamics and (**c**) biomolecular conditions. PC: protein corona: IP: isoelectric point. The figure was partly generated using Servier Medical Art by Servier, licensed under the Creative Commons Attribution 3.0 Unported Licence.

Solution conditions mainly include ionic strength (which depends on the surface charge of NP), pH, and temperature (Figure 3a).

At high salt concentrations (e.g., phosphate-buffered saline (PBS)), a higher rate of protein absorption onto NPs has been demonstrated. This phenomenon has a crucial negative drawback: NPs aggregation [10]. Indeed, it was shown that SiO_2_-NPs aggregated in buffered solutions only in the presence of bovine serum albumin (BSA), suggesting that increased ionic strength (acetate buffer (25 mL 0.2 M sodium acetate to 225 mL 0.2 M acetic acid) compared to water at pH 3.7 and PBS (137 mM NaCl, 2.7 mM KCl, 8 mM Na_2_HPO_4_, 2 mM KH_2_PO_4_) compared to water at pH 7.4) enhanced the attractive forces between BSA-NP complexes [37]. However, when the concentration of adsorbed proteins exceeds a certain threshold, the NPs achieve good colloidal stability [10]. The ionic strength also affects the surface charge of SiO_2_-NPs, which affects PC formation, as previously described. Briefly, the zeta potential of SiO_2_-NPs increased dramatically in 12 mM Na_2_HPO_4_ buffer and in PBS (zeta potential: −22.6 in water; −36.6 in Na_2_HPO_4_ buffer; −27.9 in PBS), indicating the adsorption of negatively charged ions on the particles [37]. The same variation was reported for pH, the lowering of which (from 7.4 to 6.0) was shown to drastically affect the charge of BSA-coated solid lipid NPs (SLNPs) and increase their aggregation because of the more positive charge of BSA at lower pH [38]. Therefore, pH seems to affect the ability of NPs to aggregate, adsorb proteins, and remain stable; when the solution reaches the IP value, the unstable proteins tend to either form aggregates or bind to the NPs. The pH is also a fundamental parameter to consider due to its critical role in conformational changes of certain proteins, including antibodies (Abs), which may lead to subsequent loss of functionality [39].

Temperature is an extrinsic parameter that drastically alters the composition of PC, since it has a great impact on the proteins present in biological fluids, such as serum and plasma. Indeed, heating serum to 56 °C for 30 min is a routine procedure used to induce C denaturation, which is detrimental to the PC formation onto PS-NPs [40]. Weiss et al. demonstrated that heat inactivation of plasma prevents the interaction of NPs with immune cells [41]. However, this concept is not applicable to all types of NPs. For example, Galbiati et al. showed that the pre-incubation of silver NPs (AgNPs) with heat-inactivated serum increased the production of IL-8 from macrophages [42]. It has also been demonstrated that temperature is important for the PC formation and subsequent uptake by cells. When the PC was formed at 37 °C compared to 4 °C, Lutensol-stabilized PS-NPs and cetyltrimethylammonium chloride (CTMA)-stabilized amine PS-NPs showed increased uptake into cells. An opposite trend was observed for sodium dodecyl sulfate (SDS)-stabilized carboxyl PS-NPs. This temperature-dependent uptake diversity is related to the composition of PC, which varies with temperature. Thus, a lower amount of lipoproteins was detected at 4 °C, whereas coagulation proteins were enriched. However, the dependence on temperature is closely related to the studied NP [43].

Protein absorption occurs a few seconds after NPs come into contact with biological fluids, so rapid associations and dissociations or even formation of irreversible aggregates may occur [10]. When NPs are injected in vivo, they must withstand the conditions of blood flow and navigate through blood vessels that have tortuosity, permeability, and specific hydrodynamics. Shear stress of blood vessels and other body fluids (e.g., extracellular matrix, interstitial fluid) can lead to more rapid formation and change of PC (Figure 3b) [44,45,46]. In vitro analyses must consider the dynamics of blood flow. An example of this is AuNPs that aggregate both under static incubation conditions, mainly due to the high ionic strength of the medium in which they are immersed, and under flow. However, the latter incubation condition was shown to alter the structure of PC and increase the content and abundance of adsorbed proteins [44]. This pattern was also observed for lipid and mesoporous silica NPs: dynamic flow had no effect on aggregation of NPs, but accelerated their association with proteins [45,46].

Finally, it has been demonstrated that biomolecular conditions related to the properties and concentration of proteins present in the fluid affect the formation and composition of PC. Regarding protein concentration, several literature studies have shown that the injection of metallic NPs in vivo leads to the formation of a PC composed mainly of highly concentrated proteins such as albumin, Apos, Igs, C components [47,48,49], and fibrinogen [50]. However, there is no consensus on this issue; in fact, the PC is sometimes enriched with proteins that are present in lower concentrations [51].

Among the factors affecting the identity of bound proteins, interspecies differences play an important role, especially in the preclinical to clinical perspective. This aspect became evident when studying the interactions of polymeric (dextran (DEX)- or hydroxyethyl starch (HES)-functionalized PS-NPs) and inorganic NPs (PS-NPs) with human, mouse, rabbit, and sheep plasma. First, this difference depends on the variation in the total number of proteins reviewed that are present in each plasma. This amounts to ~20,000 in human, ~16,000 in mouse, ~1000 in rabbit, and ~500 in sheep. Second, each plasma composition may affect the identity of adsorbed proteins differently (Figure 3c). Although albumin is the most abundant protein in all species, the amount of Igs varies widely (1% in mouse PC, 13% in human PC, and 7% in rabbit PC). Due to the direct role of Igs in the interaction of NPs with the immune system, their quantification is essential, further emphasizing the need for comparative experiments between biological fluids of different origins [50]. This was confirmed by Fedeli et al., who incubated SiO_2_-NPs with fetal calf serum (FCS) or HP and showed that the latter strongly reduced the internalization of NPs in macrophages, whereas FCS did not [52]. The contribution of the species to the enrichment and identity of adsorbed proteins was also confirmed for biodegradable NPs, such as those composed of poly (lactic-co-glycolic acid) (PLGA). Indeed, after incubation of PLGA NPs with human serum (HS), a greater number of proteins (~56) were identified than with fetal bovine serum (FBS, 22 to 36 proteins). An interesting enrichment of proteins involved in the immune response was found in human PC compared with bovine serum. Indeed, human PC is mainly composed of Igs fractions and C proteins (i.e., C1r and C1s). According to the authors, this result is due to the absence of antibodies in FBS, highlighting the importance of selecting the correct incubation medium when studying the PC formation [53].

The evaluation of intrinsic and extrinsic parameters together with the characterization of the PC remains essential to better predict the fate of NPs after in vivo administration. In particular, the involvement of the PC in the interaction of NPs with the immune system needs to be investigated.

## 3. The Interaction of Nanoparticles with Components of Immune System

The immune system of higher vertebrates consists of various specialized cells and soluble molecules (e.g., C) distributed throughout the body; some components circulate in the blood and lymph, whereas others are concentrated in the lymphoid organs. In general, the components of the immune system are divided into two groups: innate and adaptive immunity. These two systems work together to recognize and remove foreign materials, including engineered nanomaterials, that may share some characteristics (e.g., size and molecular surface patterns) with microbes. When the immune system fight off NPs, two different processes can be triggered: tolerance or activation. If tolerated, NPs are eliminated without triggering an immunological response; for example, via the kidneys. When not tolerated, NPs can trigger inflammation, or downregulate or upregulate immune cells [54]. Therefore, studying the interactions of NPs with the immune system is essential, especially when NPs are covered by proteins that mediate or prevent this process. Among them, two types of proteins can be distinguished: opsonins and dysopsonins.

### 3.1. Opsonins

Opsonins are proteins that reduce the half-life of NPs and accelerate their degradation in vivo by activating the immune system. In fact, the term “opsonin” refers to any substance that binds an antigen to enhance its uptake by phagocytosis. The most important opsonins are components of the C system, Igs (i.e., IgG, IgA, and IgM) and fibrinogen.

#### 3.1.1. The Complement System

The C system is the body’s first defense against foreign substances such as viruses, bacteria, and also NPs. The C cascade includes about 30 secreted factors that act alongside and in conjunction with other immune mechanisms. It consists of three activation pathways: the classical, the alternative, and the lectin pathway [55,56].

**Figure 4:**
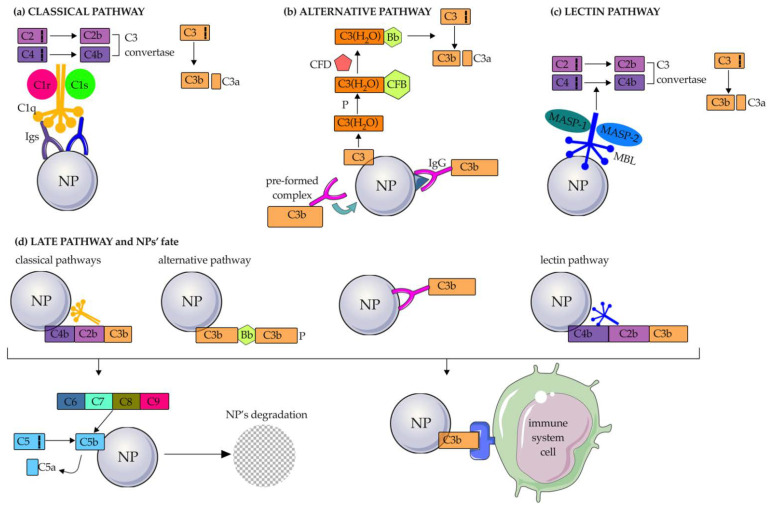
Activation of the complement system on NPs. Schematic representation of the complement activation on the surface of nanoparticles through the (**a**) classical, (**b**) alternative, and (**c**) lectin pathways till the late pathway (**d**). Igs: immunoglobulins; P: properdin; CFB: complement factor B; CFD: complement factor D; MASP: mannan-binding lectin-associated serine protease; MBL: mannose-binding lectin; ag: antigen. The figure was partly generated using Servier Medical Art by Servier, licensed under the Creative Commons Attribution 3.0 Unported Licence.

Briefly, the classical pathway is triggered by the adsorption of IgG or IgM whose Fc moieties are bound by C1q in the complex C1q-C1r-C1s (called C1), the first component of this process. Activated C1 cleaves C4 into C4a and C4b, generating a complex that then activates C2 into C2a and C2b. Consequently, the C4b2b complex (also called C3 convertase of the classical pathway) thus formed cleaves C3 into C3a and C3b (Figure 4a) [48,57]. The classical C pathway has been shown to be activated on various types of NPs, such as cubosomes [58], single-walled carbon nanotubes (SWCNTs) and hydroxyapatite NPs (HAP-NPs) [59], and poly(2-methyl-2-oxazoline) (PMOXA)-coated NPs through the deposition of C1q with an affinity comparable to that of its natural ligands [48].

The alternative pathway is characterized by a low level of constitutive activation through a process known as “tick-over” [60]. Briefly, spontaneous hydrolysis of a thioester bond formed by water molecules converts C3 to the bioactive form C3(H_2_O) (Figure 4b). In this way, the consequent structural reconfiguration of C3, which is also promoted by the presence of properdin (also known as Factor P), leads to the exposure of a binding site for C factor B (CFB). The C3(H_2_O)-CFB complex is then cleaved by a serine protease, known as C factor D (CFD), allowing the formation of the C3 convertase of the alternative pathway (C3bBb). This in turn can interact with and cleave native C3 molecules to form C3a and C3b. Liposomes made of neutral phospholipids [61], superparamagnetic iron oxide (SPIO) nano worms coated with DEX, PEGylated liposomal doxorubicin (LipoDox™), non-PEGylated liposomal irinotecan Onivyde [62], double-walled CNTs (DWCNTs), and SiO_2_-NPs mainly activate the alternative C pathway. In particular, SiO_2_-NPs have been shown to adsorb CFD and CFB to mediate this activation process. Modification of NPs with negatively charged hydroxyl and methyl groups can also activate the alternative C pathway, as C1q is not adsorbed [59]. The alternative pathway can also be triggered by IgGs adsorbed to NPs by two hypothetical mechanisms: (1) IgGs bind antigens present on the PC and are in turn attacked by pre-formed C3b; (2) an IgG-C3b complex is formed and binds adsorbed proteins to the NP surface [62].

Finally, the lectin pathway is initiated by the binding of MBL to the mannose present on an antigen or an Ig, thanks to MBL-associated serine proteases (MASP1 and MASP2), similar to C1r and C1 associated with C1q in the classical pathway (Figure 4c). This binding triggers a cascade reaction in which C4 and C2 are activated, leading to cleavage of C3 into C3a and C3b (in the same way as in the classical pathway) and forming the so-called C3 convertase of the lectin pathway.

All three C activation pathways end with the same common component: C3b. Once C3b is formed, it can bind directly to C receptors 1, 2, 3, and 4 (CR1, CR2, CR3, and CR4) expressed on monocytes and macrophages (CR1, CR3, CR4), neutrophils (CR1, CR3, CR4), B-cells (CR2), and dendritic cells (DCs) and natural killer (NK) cells (CR3 and CR4). In addition, C3b continues the C cascade by cleaving C5 into the bioactive fragments C5a and C5b. The union of C5b and other C proteins (C6, C7, C8, C9) leads to the formation of the membrane attack complex (MAC), which causes the destruction of the foreign material, including bacteria, viruses, and NPs (Figure 4d). Meanwhile, C3a, C4a, and C5a act as inflammatory mediators.

#### 3.1.2. Immunoglobulins

**Figure 5:**
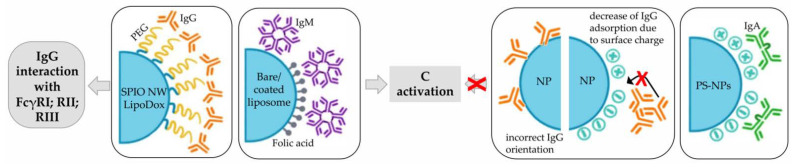
Schematic representation of the role of Igs when adsorbed on NPs. FcR: Fc receptor; SPIO NW: superparamagnetic iron oxide nanoworms; Ig: immunoglobulin; C: complement; NP: nanoparticle; PS: polystyrene.

Among plasma proteins, the major Ig classes consist of IgG, IgA, and IgM, which have concentrations of approximately 10 g/L, 2.6 g/L, and 1.5 g/L, respectively [63]. As described previously, IgG and IgM are mainly responsible for the activation of the C system; this is also true when they adsorbed to NPs. The higher efficiency of this process was further confirmed by the demonstration that the deposition of only a few Igs on the surface of NPs was sufficient to mediate C activation. Anyway, the ability of NPs to perform this process depends on the following factors: the class of adsorbed Ig, which in turn is closely related to the type of particles considered; the surface modification of NPs; and the orientation of adsorbed Abs. Specifically, C3 opsonization of SPIO nanoworms and PEGylated liposomal doxorubicin (LipoDox^®^) was predominantly triggered by the adsorption of IgGs [62], while IgM showed higher affinity for bare and folic-acid-functionalized liposomes [64]. For what concerns the effect of surface modifications on the adsorption of IgG, bare PS-NPs showed higher affinity for this Ig class than NH_2_-modified particles, and significantly more than COOH-PS-NPs. This was probably due to the formation of a large number of hydrophobic interactions or less structural rearrangements of IgG with unmodified PS-NPs, while electrostatic interactions were more dominant on modified-NPs [63]. The type of adsorbed Ig is not the only factor affecting C activation on NPs; in fact, the orientation of Abs is also of fundamental importance. Indeed, C activation can be reduced when the Fc region, the part of Abs responsible for C binding and activation, is not available due to interaction with NP (Figure 5) [62].

Along with C activation, it has been shown that adsorption of IgG to charged NPs dramatically increases their aggregation, which can lead to immune system responses [63]. Fortunately, this is not the rule. Even though IgGs induced more aggregation when compared to other proteins (e.g., fibronectin, HSA, and Apo-A1), they confer colloidal stability at high concentrations when coated on AuNPs [65].

Only a limited amount of information has been published about IgA in the literature. In a recent article by Prozeller et al. it was shown that IgAs can bind bare, as well as negatively and positively charged-PS-NPs [63].

The role of Igs is not limited to activation of C pathways, but they can also directly interact with receptors expressed on immune cells and cause rapid clearance of NPs to which they are adsorbed. In particular, bound IgG target FcγRI (CD64) expressed on phagocytes, FcγRII (CD32) present on B-cells, monocytes/macrophages, and granulocytes, FcγRIIIa (CD16) expressed on macrophages, NK cells and a subset of T cells [66].

#### 3.1.3. Fibrinogen

**Figure 6:**
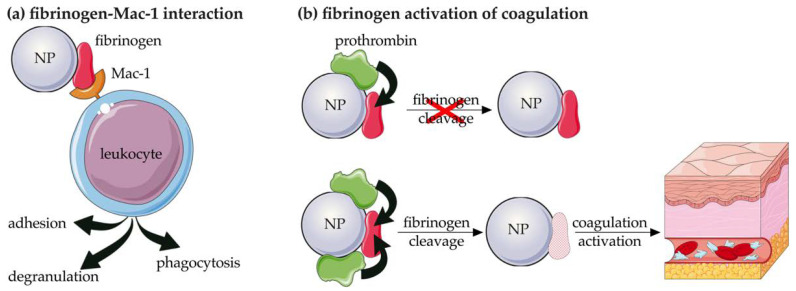
Schematic representation of the mechanisms mediated by the deposition of fibrinogen on NPs. (**a**) interaction with Mac-1 receptor expressed by leukocyte and (**b**) direct lysis of fibrinogen. NP: nanoparticle. The figure was partly generated using Servier Medical Art by Servier, licensed under the Creative Commons Attribution 3.0 Unported Licence.

Fibrinogen is a 340 kDa plasma glycoprotein involved in the blood coagulation process through its conversion to fibrin via a mechanism mediated by thrombin, which is the major event in blood clot formation. Fibrinogen also plays a key role in the crosstalk between coagulation and inflammation by triggering leucocyte activation. This process is mediated by the interaction between fibrinogen and the fibrinogen-binding receptor Mac-1, which is expressed on activated leukocytes (i.e., neutrophils and monocytes) and promotes adhesion, phagocytosis, and degranulation (Figure 6a). The interaction is significantly enhanced when fibrinogen is in an unfolded state, a condition that is present when it adsorbed to negatively charged poly(acrylic acid) (PAA)-conjugated Au-NPs, resulting in increased immunotoxicity of these nanostructures [67].

Among its multiple ligands, Mac-1 also directly binds C components such as iC3b and Factor H (CFH), a degradation product of C3 and a regulatory protein of the alternative activation pathway, respectively, further highlighting the close interaction between coagulation and inflammation [29]. This connection is further confirmed by the ability of coagulation components (i.e., thrombin, Factor Xa, plasmin, and kallikrein) to strongly activate C, after activation of the coagulation cascade during inflammation [68,69] by cleavage of C3 and C5, and resulting in a time- and concentration-dependent release of C3a and C5a in vitro [69].

The procoagulant activity and immunotoxicity of NPs are related to the cleavage of the adsorbed fibrinogen as the first step in the coagulation process and depend on the type of nanostructure considered. In fact, it has been demonstrated that the deposition of fibrinogen on PAA-zinc oxide (ZnO) and PAA-capsules stimulates its cleavage and activation [70]. On the contrary, some types of elastin-like polypeptides (ELPs) have been shown to activate platelets due to fragmentation of deposited fibrinogen. The cleavage of fibrinogen was also dependent on the amount of adsorbed prothrombin [71] (Figure 6b). Fibrinogen was also deposited on metal oxide [72] and titanium dioxide (TiO_2_) NPs [73], being the most abundant adsorbed protein. Moreover, positive coagulation regulators of fibrinolysis were more strongly associated with metal-oxide NPs than negative ones [72].

### 3.2. Dysopsonins

The term “dysopsonins” refers to any substance capable of inhibiting immune system activation and phagocytosis through a phenomenon known as “*stealth*”. Dysopsonins include histidine-rich glycoprotein (HRG), Apos, HSA, and surfactant protein A and D (SP-A, SP-D).

#### 3.2.1. HRG

HRG is the major component of human and mouse SiO_2_-NPs PCs. In fact, HRG accounts for 23% of PC adsorbed on acrylic acid (AC)-coated SiO_2_-NPs (zeta potential: −29 mV), 16% of proteins deposited on methyl-oxazoline (OX)-coated SiO_2_-NPs (zeta potential: −18 mV), 12% of PC on 1,7-octadiene (OD)-coated SiO_2_-NPs (zeta potential: −16 mV) and 13% of proteins deposited on positively charged (zeta potential: +2 mV) allylamine (AA)-coated SiO_2_-NPs. These results also indicated the preferential binding of HRG to strongly negatively charged SiO_2_ NPs [51]. Adsorption of HRG has been shown to confer stealth properties to NPs against engulfment by macrophages (Table 1) [51,52]. However, this process is limited by the dose of injected NPs. Notably, HRG shows high affinity for SiO_2_-NPs when injected at a low dose; however, as the dose of NPs increases, HRG is exhausted from the plasma, leaving an empty space on the surface of NPs, which becomes suitable for the recruitment of other proteins with lower affinity [23].

#### 3.2.2. Apolipoproteins

Apos are a class of proteins that bind lipids to transport them. In the context of the interaction between NPs and the immune system, Apos are known to modulate C activity; in particular, high-density lipoproteins (HDL, Apo-AI) and, to a lesser extent, Apo-AII inhibit C9 polymerization, thus hindering the formation of MAC [78]. Moreover, adsorption of Apo-AI has been shown to prevent macrophages from engulfing liposomes, in an abundance-dependent way and with a strong positive correlation with the lifetime of NPs in the systemic circulation [75]. Apo-J plays a similar role by inactivating C5b-9 complexes in cooperation with vitronectin [79]. The role of Apo-J is not limited to direct inhibition of C components, but it also attenuates opsonization of NPs by Igs, which in turn reduces uptake by phagocytes and clearance of NPs [13]. At physiological concentrations, Apo-J reduced the uptake of PS-NPs and PEGylated polymeric NPs by macrophages by 75.4%; in contrast, a higher percentage of uptake was observed in the absence of Apo-J, confirming previous reports. Additionally, the stealth properties of polymeric NPs are not entirely related to PEG itself, but rather to its ability to prevent or completely abolish the deposition of Apo-J on the NPs [14]. The same pattern has been evidenced for SiO_2_-NPs and AgNPs (Table 1) [74].

#### 3.2.3. Albumin

Albumin has been frequently identified in the PC of various types of NPs, modulating their tissue localization and cell targeting. It is present in high concentrations in human blood (35–50 g/L) [80] and is considered an excellent NP carrier thanks to its non-toxicity and non-immunogenicity. In addition, albumin possesses both -COOH and -NH_2_ groups that facilitate its functionalization, which explains why it is often used to engineer NP coating to improve stealth properties and drug delivery [81]. In fact, incubation of PS microparticles and nanospheres with HSA has been shown to strongly reduce phagocytosis by DCs, even in the presence of opsonins such as IgG and α2 human serum glycoprotein [76], and hepatic uptake [82], respectively. Coating hyaluronic acid (HA)-NPs with HSA has been shown to increase circulation time compared to free drugs and improves the delivery of the payload (i.e., Erlotinib, ERT) [77]. This strategy also appears to be reproducible for albumin from other species; in fact, a preformed corona of BSA on poly-3-hydroxybutyrate-co-3-hydroxyhexanoate (PHBHHx) NPs has been shown to reduce adsorption of IgG and C components in rats, resulting in a significant decrease in the clearance speed [74] (Table 1). However, there is no consensus in the literature on the dysopsonic nature of albumin, mainly due to the fact that most studies were performed with purified protein, which is not representative of the real concentration in biological fluids [14].

#### 3.2.4. SP-A and SP-D

SP-A and SP-D are two hydrophilic proteins that belong to the collectin family and represent the main component of the pulmonary surfactant. In this organ, their role is to prevent bacterial infections by interacting with other immune agonists, such as C1q and IgA [23]. Because of these properties, SP-A and SP-D usually favor the interaction of NPs with phagocytes; however, this is not the rule. In fact, SP-A acts as a dysopsonin when adsorbed to anionic PS-NPs, inhibiting their uptake by alveolar macrophages both in vitro and in vivo [83].

As for SP-D, it has been shown to downregulate the interaction of oxidized CNTs (Ox-CNTs) with macrophages, confirming its dysopsonic nature (Table 1) [23].

As mentioned above, all proteins described in this section are considered dysopsonins, but for some of them (i.e., HSA, Apo-AI, SP-A, and SP-D) this definition strictly depends on the nanostructures studied. Indeed, HSA and Apo-AI alone and in combination have been shown to increase the uptake of SiO_2_-NPs by human macrophages compared to the serum-free condition [84]. SP-A acts as an opsonin by significantly increasing the uptake of cationic PS-NPs [83] and magnetic NPs coated with chitosan, polymaleic-oleic acid (PMO) and phosphatidylcholine (PL) in mouse alveolar macrophages [85]. The same pattern was confirmed for mannose-modified PLGA/poly lactic acid (PLA)/PEG NPs which were internalized within human macrophages, both in vitro and in vivo, to a much higher extent in the presence of SP-A. Moreover, this effect was found to be dependent on mannose concentration, suggesting direct binding of this sugar to SP-A [86]. On the other hand, SP-D adsorbed on Ox-CNTs and carboxymethyl cellulose (CMC)-CNTs was shown to significantly enhance the C activation compared to uncoated CNTs and provide more sites for covalent binding of C3b and C4b to NPs. However, only the uptake of CMC-CNTs by macrophages was increased by the adsorption of SP-D, whereas Ox-CNTs showed an opposite pattern, as mentioned previously [87]. The opsonic nature of SP-D was also suggested in vivo: macrophages purified from SP-D^-/-^ mice showed reduced uptake of naked PS-NPs, amino PS-NPS, and carboxylate PS-NPs compared to normal cells [88].

### 3.3. Innate Immune System

The innate immune system is the first effective defensive mechanism against pathogens. It has the role of scanning the body to remove apoptotic bodies, protein aggregates, and cellular debris; it also directly eliminates pathogens and abnormal cells. The importance of PC in this field lies in its ability to be directly recognized by the innate immune system, leading to rapid elimination of NPs by the liver [89,90,91,92], spleen [93], and lungs, organs in which phagocytic cells are localized. This recognition is mediated by receptors such as scavenger receptors (SRs), FcγR and CRs expressed on the surface of immune cells, resulting in the phagocytosis of NPs and their rapid elimination from the bloodstream [94]. Cells of the innate immune system include monocytes, NK cells, polymorphonuclear (PMN) leukocytes (i.e., neutrophils, eosinophils, basophils, and mast cells), and DCs [54]; cells belonging to the so-called mononuclear phagocytic system (MPS) are mainly responsible for the elimination of NPs through the recognition of PC opsonins [95]. The MPS is composed of macrophages and DCs that are found in various organs and tissues, particularly in reticular connective tissue, liver, spleen, and lymph nodes. Because of their central role in the clearance of nanomaterials, most research has focused on monocytes and tissue-derived macrophages. Regarding other innate immune cells, the response of PMN cells, other than neutrophils, and NK cells to nanomaterials has been poorly studied; no published articles on this topic could be found.

#### 3.3.1. Macrophages

Macrophages are cells of the innate immune system localized in all tissues, especially those with a barrier function (mucosae—both digestive and respiratory—and skin), and are involved in the clearance of pathogens, as well as nanomaterials [96,97], wound repair, and homeostasis.

In mammals, macrophages are divided into three distinct populations: M0 macrophages, which have not received stimulation and are in an inactive state; M1 macrophages, which are active in pro-inflammatory processes; and M2 macrophages, which are active in anti-inflammatory or degenerative processes (e.g., wound healing; cancer, diabetes, etc.) [98]. In general, macrophages can recognize a wide range of endogenous and exogenous ligands by expressing a variety of receptors: receptors for Igs (FcR), SRs (e.g., SR-A and CD36), Toll-like receptors (e.g., TLR2 and TLR4), CD14, CRs (e.g., CR3 and C5aR), immunoreceptor tyrosine-based activation motif (ITAM), chemokine receptor type 2 (CCR2), epidermal growth factor (EGF)-like module-containing mucin-like hormone receptor-like 2 (EMR2), Dectin-1, DC-specific intercellular adhesion molecule (ICAM)-3-grabbing non-integrin (DC-SIGN), and myeloid mineralcorticoid receptor (MR) [99]. As described previously, dysopsonins and opsonins are key factors that influence the interaction of NPs with immune cells directly through these receptors, particularly FcRs, SRs and TLRs.

**Figure 7:**
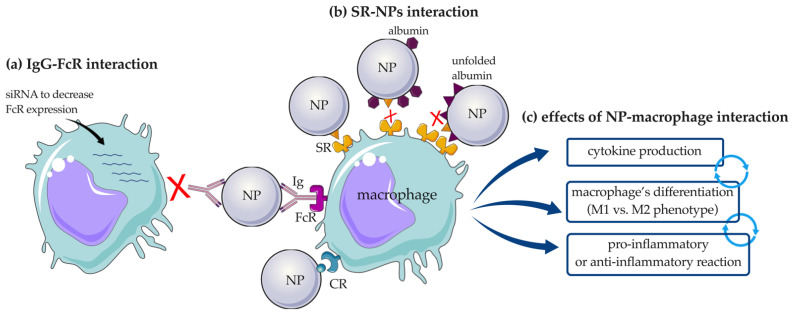
Schematic representation of the interaction of NPs with macrophages. (**a**) Involvement of the Ig-FcR interaction, and (**b**) role of the adsorbed proteins in the interaction with SRs. (**c**) effects of NP–macrophages interaction; NP: nanoparticle; IgG: immunoglobulin G; FcR: Fc receptor; SR: scavenger receptor; CR: complement receptor. The figure was partly generated using Servier Medical Art by Servier, licensed under the Creative Commons Attribution 3.0 Unported Licence.

It has been shown that PC influences the uptake of NPs by a specific subset of macrophages depending on the expression of such receptors. For example, SR-A and mannose receptor CD206 were have been shown to be involved in the uptake of uncoated mesoporous SiO_2_-NPs by macrophages. The involvement of CD206 in the uptake of mesoporous SiO_2_-NPs by proinflammatory macrophages was highlighted by pre-treatment of cells with mannan, a competitive ligand of the mannose receptor, which significantly reduced this interaction. The same result was obtained by silencing CD206 on pro- and anti-inflammatory macrophages after stimulation with granulocyte macrophage colony-stimulating factor (GM-CSF) and macrophage colony-stimulating factor (M-CSF), respectively [100]. To demonstrate the importance of the involvement of PC in the interaction of NPs with macrophages in different activation states, a study was performed using SiO_2_-NPs of different sizes (50, 100, 200, 500, and 1000 nm). Larger SiO_2_-NPs (diameters of 500 nm and 1000 nm) were preferentially engulfed by M1 macrophages compared with M2 macrophages under serum-free conditions, whereas an opposite pattern was observed when they were pre-incubated with serum. This difference was explained by the expression of CR3 and FcγRII by M2 macrophages, which can interact with the proteins on PC, while they are absent on the M1 population [101]. Further studies investigating the involvement of FcRs in the engulfment of PS-NPs by human macrophages involved the addition of antisense oligodeoxynucleotides, which specifically reduced FcγRI expression. This pretreatment was shown to inhibit the internalization of PS-NP by macrophages, confirming the importance of IgG adsorption, which is the major protein group detected, and its interaction with the FcγRI receptor (Figure 7a) [102].

SR, and in particular SR-B1, are known to mediate the uptake of AgNPs with subsequent accumulation in macrophages and apoptosis, release of the inflammatory cytokine oncostatin M, and inhibition of the CD68 surface marker responsible for cell activation. It was shown that the coating with proteins such as Apo-AI and albumin prevented immunotoxicity of AgNPs and reduced SR-B1 uptake by macrophages [103]. The same pattern became visible when the macrophage receptor with collagenous structure, MARCO, belonging to the SRs family, was studied in terms of its interaction with SiO_2_-NPs. In fact, the addition of unfolded BSA to HS-incubated SiO_2_-NPs inhibited their interaction with MARCO [104]. With respect to the dysopsonic nature of unfolded albumin, contrasting results have been published. One example is the conformational changes that albumin undergoes after adsorption onto silicate NPs [105], PEGylated CNTs [106], and nanoporous polymeric NPs [107], making the nanostructures more easily recognized by SR-A expressed on macrophages [105] (Figure 7b) with consequent triggering of the immune response and secretion of interleukin (IL)-1β and tumor necrosis factor alpha (TNF-α) [106]. Cytokine release is a marker of macrophage activation, and it has therefore been widely studied. Six different HP-coated polymeric NPs (consisting of nanogels and colloidal NPs) were incubated with primary human macrophages and compared with pristine NPs. HP-coated nanogels were found to increase the release of IL-6 and IL-10, whereas IL-1β was not affected. However, neither the corona-bearing nor pristine colloidal NPs affected cytokine production. Although IL-10 has a known immunosuppressive role, its overexpression could possibly indicate the immunogenicity of the NPs, as its production normally corresponds with the release of other pro-inflammatory cytokines to regulate the inflammatory process [108]. In contrast, the release of IL-1β was detected after incubation of macrophages with Au-nanorod surrounded by PC and correlated positively with proteins involved in tissue leakage, acute phase, and C activation [9]. The evidence that proteins adsorbed on NPs can exert proinflammatory activity on macrophages is also supported by the study of Mo et al., who showed that plasma-coated black phosphorus nanomaterials (BPQDs) significantly increased the release of inflammatory cytokines, including IL-1β, IL-6, IL-8, and interferon (IFN)-α [109].

The PC may not only affect the interaction of NPs with receptors expressed on macrophages or trigger cytokine production; it can also influence the polarization of the cell response (Figure 7c). Exposure of macrophages to bare AuNPs or AuNPs preincubated with HP accurately demonstrated this theory. Increased release of IL-1β and TNF-α from macrophages was detected after a long incubation period (12 h) of HP-AuNPs, indicating differentiation of macrophages into the M1 phenotype. In contrast, a shorter incubation time (4 h) of HP-NPs resulted in a significant increase in IL-10 production, a clear sign of differentiation toward the M2 phenotype. This difference is related to the change in identity of adsorbed proteins; in particular, Igs, C components, fibrinogen, and haptoglobin, which are associated with the immune response, have been shown to increase with time, influencing macrophage polarization [110].

#### 3.3.2. Dendritic Cells

DCs are professional antigen-presenting cells (APCs) that enable the interplay between the innate and the adaptive immune responses. Immature DCs reside in non-lymphoid tissues and undergo a maturation process in the presence of stimuli (e.g., allergens, cytokines, bacteria). They then migrate to the lymph nodes, where they activate T lymphocytes. Besides macrophages, DCs are the most studied cell type in NPs clearance research due to their key role in the immune system. DCs localized in secondary lymphoid tissues have been shown to be frequently bound by NPs-based vaccine approaches, suggesting their involvement in the clearance of such structures. Some of the most common receptors expressed on DCs are C receptors (CR3/4), pattern recognition receptors (PRRs), which include TLRs, cell surface C-type lectin receptors (CLRs) and intracytoplasmic nucleotide oligomerization domain (NOD)-like receptors (NLRs), and FcR [111]. Among them, CRs and PRRs have been best characterized. Bednarczyk et al. have demonstrated the contribution of CRs to the internalization of NPs in DCs through a C-dependent mechanism. The authors preincubated solid iron oxide (FeO)-DEX and carbohydrate-coated NPs (i.e., bionized nanoferrite (BNF)-DEX and BNF-starch NPs) with mouse serum, which led to the deposition of C on their surfaces. Bone-marrow-derived DCs (BMDCs) expressing CR3 and CR4 were shown to bind native serum-pretreated NPs to a higher extent than untreated or heat-inactivated serum-pretreated NPs. Further characterization in CR3^-/-^ mice revealed that only CR3 is required for the binding of C-opsonized NPs, whereas CR4 is not. The resulting interaction of NPs also affected the expression of the major histocompatibility complex II (MHCII) and the costimulator CD86, both of which were decreased, resulting in reduced responsiveness to stimulation with LPS [112].

As for PRRs, they have been shown to be involved in the internalization of PLGA-NPs in DCs. This mechanism was particularly promoted by the presence of lipopolysaccharide (LPS), a natural environmental contaminant, in a batch of NPs after manufacturing. If not properly detected, LPS can modulate the immune response to NPs, falsifying in vitro and in vivo results. This is due to the property of LPS to be recognized by TLRs and NLRs expressed on DCs and to induce antigen-specific CD8^+^ T cell responses [113].

#### 3.3.3. Neutrophils

Although neutrophils play a key role in inflammatory processes and have been shown to infiltrate tissues after exposure to NPs, few studies have been published on their importance in nanotoxicology. Neutrophils are the first cell type to respond to inflammation, and they are now known to be directly involved in the clearance of NPs in some strains of mice. Neutrophils are removed from the bloodstream by the liver and spleen and, in particular, by the bone marrow, which consequently can be considered a site of sequestration of NPs [114].

It has been demonstrated that neutrophils in the blood are involved in the clearance of carbohydrate DEX-coated NPs. This process is mediated by the activation of the C pathway through the deposition of C3 on NPs and CR3 on the cell surface. However, this is not true for all DEX-coated NPs; other contrasting studies showed the involvement of SR-A but not CR3 in this process (Table 2) [112].

PEGylation is a common method used to significantly reduce the interaction of NPs with macrophages, but this is not true for neutrophils; in fact, PEGylated PS microspheres have been shown to be more internalized by neutrophils in the presence of HP [114]. The same was reported for SWCNTs, which also affected degranulation, myeloperoxidase (MPO) release [114,115,116], and intracellular reactive oxygen species (ROS) production after adsorption of IgG on their surface. In contrast, PEG-SWCNTs treated with HSA showed a slight decrease in neutrophil activation [114,115].

### 3.4. Adaptive Immune System

The adaptive immune system is recruited directly from the innate immune system to induce a more specific defense, and provides slower but more efficient protection against pathogens [54]. Adaptive immunity has been shown to be directly or indirectly involved in NP clearance. Specifically, the direct mechanism involves the interaction and elimination of NPs by cells of the adaptive immune system, such as B and T lymphocytes. On the other hand, the same cells can modulate the activity of macrophages to be more likely to engulf NPs via the indirect mechanism [117].

The direct interaction between NPs and adaptive immune cells is confirmed by PEG-conjugated nanoformulations, which have been shown to elicit adaptive immune responses through anti-PEG antibodies [118,119]. Moreover, recent literature studies report that the adaptive immune system can rapidly recognize NPs when unfolded or aggregated proteins are present on them [106,120].

Little research has been conducted on the interaction of other types of NPs, especially polymeric NPs, and their PCs with the adaptive immune system.

#### 3.4.1. B Lymphocytes

B lymphocytes are white blood cells responsible for humoral immunity, a component of the adaptive immunity mediated by molecules such as Abs secreted in extracellular fluids. Among B lymphocytes, B-1 cells constitute a smaller B cell population that express CR1/2 and CR3 and are thus able to bind activated C components. With respect to the formation of PC on NPs, B-1 cells were shown to bind FeO-DEX NPs to a greater extent than conventional B-2 cells after incubation with serum. Moreover, C-opsonized NPs were bound only through the CR1/2 receptors (Table 2). The role of C deposition on NPs in this process was further emphasized by the complete abolishment of the interaction between B-1 cells and particles after serum heat inactivation [112]. This interaction was also confirmed in vivo [77]. The uptake of FeO-DEX NPs in B-1 cells also resulted in an inhibitory effect on the expression of activation marker (lower upregulation of CD86) [112].

#### 3.4.2. T Lymphocytes

Folding of proteins bound to NPs has been shown to affect the T-cell response. Indeed, after repeated administration of CNTs coated with unfolded proteins in mice, the immune cell population in the spleen was examined, and it was found that the number of effector T helper (CD4+) cells increased, while the number of naïve CD4+ T cells decreased (Table 2) [106].

## 4. Strategies to Evade Immune System Activation

Because opsonization and removal of nanocarriers from the bloodstream by the MPS are major challenges for effective drug delivery, researchers have focused their attention over the past decade on developing new strategies to circumvent recognition by the immune system.

Due to the complexity of the immune system and its many players, evasion of the immune system is often achieved through multiple approaches that can target different levels of the immune response. Initial studies focused on reducing the binding of proteins, particularly opsonins, to NPs; over time, this trend is changing and researchers are turning their attention to exploiting PC by binding dysopsonins.

Possible methods include modifying the surface of NPs with different types of coatings (polymers, proteins, or biomimetic coatings) and reducing the number of proteins bound to the surface, e.g., by creating a protein shield or increasing the binding of dysopsonins (using proteins that promote bypassing phagocytosis) [121].

**Figure 8:**
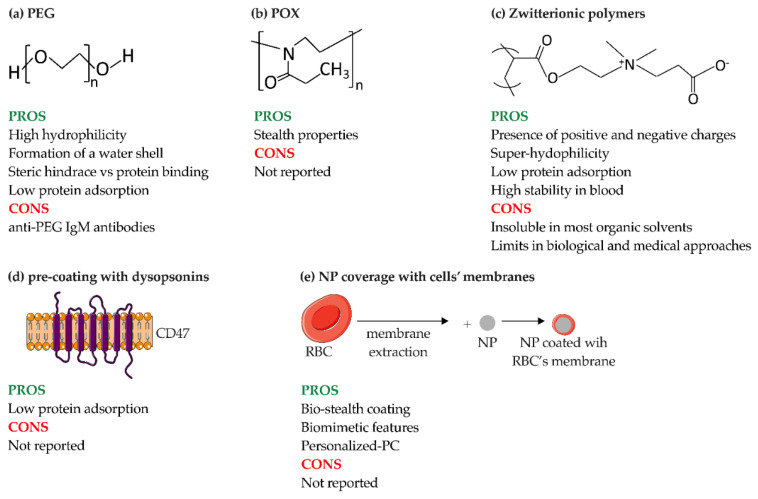
Methods to avoid immune system engulfment of NPs. (**a**) PEG; (**b**) POx; (**c**) zwitterionic polymers; (**d**) pre-coating of NPs with dysopsonins; and (**e**) coverage of NPs with membranes derived from cells. POx: poly(2-Oxazoline); RBC: red blood cell. The figure was partly generated using Servier Medical Art by Servier, licensed under the Creative Commons Attribution 3.0 Unported Licence.

One of the most common surface modifications is the addition of a polymeric coating, such as PEG (Figure 8a). The process of PEGylation provides stealth properties by forming a water shell, which is a steric hindrance and prevents protein coating on NPs. However, such repulsion requires a minimum layer thickness, which depends on the MW of PEG, its conformation and the density of the chains [36]. In 2001, Panagi et al. demonstrated that negatively charged, non-PEGylated PLGA-NPs with a size of about 154 nm are rapidly sequestered by MPS organs, especially the liver, within few minutes and in a dose-dependent manner. In contrast, their PEGylated counterpart (mPEG-PLGA-NPs, ~113 nm) was sequestered approximately 7 h after administration in a dose-independent manner, highlighting the role of PEG in prolonging the circulation time of particles in the blood [122]. Their results were also confirmed by Schöttler et al., who demonstrated that the decreased opsonization of PEG-coated PS-NPs resulted in reduced uptake by murine macrophage (RAW264.7 cells). For this purpose, PS-NPs coated with different amounts of PEG, ranging from 44 (PS-PEG_44_) to 110 units (PS-PEG_110_), were produced. After incubation with HP, the percentage of adsorbed proteins was determined: PS-PEG_44_ and PS-PEG_110_ showed a 79% and 66% reduction in bound proteins, respectively, compared with non-functionalized PS-NPs. The presence of PEG also affected the type of proteins bound, with an increase in the binding of dysopsonins, such as clusterin, which accounted for approximately 50–60% of the proteins in PC of PEGylated NPs [14]. All these advantages make PEGylated nanoformulations suitable for clinical applications. This is the case of LipoDox^®^ (PEGylated liposomes) and Oncaspar^®^ (PEGylated enzyme), which are approved for the treatment of cancer, and Pegasys^®^ (PEGylated interferon α-2a) and Pegintron^®^ (PEGylated interferon α-2b), which are approved for the treatment of hepatitis [2].

Despite these advantages, exposure to PEG can lead to the production of anti-PEG Abs, particularly IgM and IgG, which accelerate the clearance of PEGylated NPs [118]. In addition, the production of specific anti-PEG IgE or IgG can lead to the initiation of hypersensitivity or C-activation-related pseudo-allergy (CARPA) reactions, which may prevent prolonged clinical use of PEGylated NPs [123]. Thus, despite the advantages of PEGylation, sooner or later, NPs are recognized by the immune system; consequently, it is also necessary to use PEGylation in synergy with other strategies.

Other types of stealth polymer coatings are poly(2-Oxazoline) (POx) and zwitterionic polymers. POx is a hydrophilic polymer with stealth properties similar to PEG, making it a potential candidate for overcoming the limitations of PEG. Among POx, PMOXA and poly(2-ethtyl-2-oxazoline) (PEtOx) also offer advantageous properties, such as biocompatibility, thermo-responsiveness, and high stability (Figure 8b). In general, POxs are ideal candidates for clinical applications; indeed, they have been shown to be safe and stable both in vitro and in vivo, even after repeated administration [124]. Coating the surface of NPs with POx chains can improve their circulation time and give them stealth properties due to the absence of hydrogen bond donors in the polymer, making them biocompatible and resistant to protein adsorption [125]. As for PMOXA, when coated with nanostructures, it shows high hydration and low biofouling comparable to PEG coatings. These properties are closely related to the length of PMOXA chains; the longer the PMOXA chain, the farther the surface layer spreads from the core, resulting in better protein repulsion, and thus delaying the association of the polymers with macrophages and extending blood circulation time [126]. However, there is little literature on how these polymers might affect the composition of PC.

Zwitterionic polymers possess positively and negatively charged groups and, like PEG, show promising stealth properties (Figure 8c) [12,41]. Since zwitterionic polymers are superhydrophilic, they are used for applications where the hydrophobic property of PEG would be an obstacle (i.e., decreased bioactivity of proteins, NP instability, and destabilization of lipid bilayers) [127]. Another advantage over PEG is the longer stability of zwitterionic polymers, especially poly(carboxybetaine) (PCB), in blood plasma [127]. The mechanism of action of zwitterionic polymers, in addition to balanced surface charge, is based on the creation of highly hydrophilic surfaces that reduce protein adsorption [128] and thus prolong blood circulation time. Another advantage is the tunability of the surface charge of NPs composed of zwitterionic polymers. Indeed, NPs composed of poly(εcaprolactone) (PCL), poly(allyl ethylene phosphate) (PCLb-PAEP) and zwitterionic polymers with a pH-sensitive group in the anionic part are able to switch charge when exposed to an acidic pH (of 6.8), both in vitro and in vivo in an orthotopic nude mouse model of human breast cancer. In the latter context, zwitterionic polymers were able to switch to a positive charge due to the accumulation in the acidic tumor tissue resulting in enhanced uptake by tumor cells in vivo and subsequent inhibition of tumor growth [129]. However, zwitterionic polymers are insoluble in most organic solvents due to their superhydrophilic nature, which hinders their potential clinical application.

Another approach to evade the immune system is to rationally pre-coat NPs to design a PC that can reduce cellular uptake. For example, COOH-PS and NH_2_-PS-NPs were precoated with Ig-depleted plasma and then incubated with macrophages grown in medium containing or lacking plasma proteins. With particular reference to PC composition, the precoated COOH-PS-NPs were surrounded by a fibrinogen-rich protein layer (74%), whereas incubation with complete plasma resulted in the formation of a PC that was highly enriched in vitronectin (33%). However, this pattern did not affect the uptake of NPs by RAW264.7 cells. An opposite result was obtained for NH_2_-PS-NPs; their pre-coating with Ig-depleted plasma resulted in the predominant adsorption of hemopexin (38%) and clusterin (20%), whereas the incubation of uncoated NH_2_-PS-NPs with complete plasma led to the accumulation of clusterin (60%). Adsorption of this protein, albeit in lower amounts, strongly reduced the uptake of pre-coated and uncoated NPs into macrophages [130].

Similarly, several research groups have addressed the enhancement of dysopsonins (i.e., albumin, Apos, or specific ligands) bound to the surface of NP. One example of the use of specific ligands is CD47, an immunoglobulin-like protein expressed on hematopoietic stem cells and many types of cancer cells that functions as a “self” or “do not eat me” marker. A study investigating this possibility was performed by Rodriguez et al., who demonstrated a reduction in phagocytosis of streptavidin-coated PS beads conjugated with synthetic human CD47 by THP-1 cells (Figure 8d) [121]. In vivo experiments with Non-Obese Diabetic-Severe Combined Immunodeficiency-gamma (NSG) mice also confirmed the longer circulation time of CD47-conjugated NPs compared to PEGylated NPs.

In particular, with regard to cancer therapies, NPs could be cloaked with natural cell membranes to improve their targeting, increase biocompatibility, and prolong circulation time [131,132]. The translocation of the cell membrane onto the NPs masks their biochemical properties and confers stealth capability. An example of this is red blood cells (RBCs), which are commonly used as bio-stealth coatings (Figure 8e) [133]. RBC-coated PLGA-NPs associated with both lipids and surface proteins, exhibited a longer half-life than PEGylated NPs in a mouse model. These results demonstrate that RBC membranes coating confers biomimetic properties and stealth capacities to NPs, and even outperforms conventional PEG stealth coating in terms of in vivo clearance [134]. Cell membranes derived from macrophages and cancer cells are other examples of biomimetic coatings of NPs. As for macrophages, liposomes coated with RAW264.7 membranes have been shown to specifically mediate and increase the uptake of NPs in breast cancer through the interaction of integrin α4β1, which is present on the macrophages membrane, and vascular-cell adhesion molecule 1 (VCAM-1), which is expressed by cancer cells. It has also been shown that the coating of NPs with macrophage membranes greatly reduces their interaction with RAW264.7 cells, which improves the blood circulation and targeting ability of the coated NPs [135]. Reduction in the interaction of NPs with macrophages in vivo was also achieved by coating PLGA particles with a combination of cancer (4T1 breast cancer) and macrophage (RAW264.7) membranes, highlighting the importance of combining multiple strategies to reduce immune system activation and improve therapeutic efficacy [136].

## 5. Conclusions and Perspectives

The application of NPs in medicine is a rapidly expanding field of research, with emphasis on drug delivery. This interest is based on the ability of NPs to encapsulate various cargos, such as chemotherapeutic agents, thus improving the efficacy of targeted delivery while minimizing their side effects. The interaction of NPs with biological fluids, on the other hand, is a key challenge. When NPs enter the bloodstream, their surface becomes covered by proteins, leading to the formation of a PC that affects the identity and behavior of NPs. More importantly, the PC is instrumental in immune recognition by MPS cells through the binding of opsonins, leading to rapid and premature elimination from the circulation. Interestingly, proteins identified as dysopsonins were also detected in the PC of several NPs. These proteins are able to confer stealth properties to NPs, increasing circulation time and efficacy. Consequently, the delicate balance between opsonin and dysopsonin is critical for predicting the fate of NPs in vivo. Considering all this, the characterization of PC is an essential step in the development of NPs as DDS. Indeed, PC has been shown to have a strong impact on the toxicity and efficacy of NPs through several mechanisms of action: (i) as largely described in this review, PC can alter the biodistribution of nanostructures by mediating their rapid elimination by the liver after uptake by MPS cells; (ii) the PC layer formed on the surface of NPs may also shield the interactions between ligands bound on the surface of NPs and their targets [137]; (iii) an additional mechanism may be related to the action of the C system, which can induce the degradation of NPs with consequent leakage of loaded drugs.

However, characterization of PC is often carried out with simple in vitro experiments using techniques such as SDS-polyacrylamide gel electrophoresis (SDS-PAGE), which are often not very informative. On the other hand, more informative experiments, such as those performed in vivo with animal models, are often not possible. Moreover, conflicting results can be explained by differences in PC due to the biological environment in which NPs are tested. In this context, Hadjidemetriou et al. examined the PC profile of 115 nm, negatively charged Doxil^®^ liposomes 10 min, 1 h, and 3 h after injection into CD-1 mice. The PC formed as early as 10 min after administration and the composition profile remained nearly constant. Specifically, the most abundant protein at 10 min was macroglobulin, whereas Apo-E and hemoglobin predominated at 1 h and 3 h, respectively. Although these liposomes were specifically designed to have an extended half-life in the circulation, the in vivo PCs formed at the different time points included several key proteins of the C cascade involved in the classical, alternative, and lectin pathways. These results highlight the importance of understanding the biological implications of in vivo PC formation in order to rationally design NPs with improved therapeutic efficacy [138]. While a relevant amount of data has been collected on the hard corona, knowledge on the composition and biological relevance of the soft corona is rather incomplete.

Most publications have therefore focused on the interaction between NPs and macrophages or DCs, which are the main agents responsible for rapid detection and phagocytosis. Many of these studies have shown that opsonins bound to NPs, particularly Igs and the C system, play a crucial role in MPS recognition. It has also been reported that the interaction between PC and immune cells drives polarization toward specific cell types, as demonstrated for macrophages and DCs [101,112], thereby enhancing inflammation and influencing the toxicity of NP.

Compared to MPS cells, much less attention has been paid to the interaction of PC proteins with other cells of the immune system, such as PMN, NK cells, and B and T lymphocytes. These cells, in combination with MPS components, are also involved in immunological and inflammatory processes; therefore, research on the PC influence on these cells should be a priority.

Interaction of NPs with biomolecular components is inevitable; therefore, there is a growing interest in applications using engineered PCs to hide NPs from the immune system (stealth), improve their targeting ability, and use them for vaccine development. Indeed, NPs engineered to target immune cell receptors can aid in the co-administration of antigens and adjuvants to act as nanovaccines. One such receptor is CLR, which upon activation affects APC activity. Polyanhydride NPs functionalized with specific carbohydrates have been shown to target CLRs on alveolar macrophages and increase the expression of macrophage mannose receptor (MMR), MHCI, and MCHII. Under the same circumstances, increased production of the proinflammatory cytokines IL-1, IL-6, and TNF-α was observed. Therefore, targeting MMR and other CLRs could be a promising technique to improve the adjuvant effect of nano-vaccines [139].

Regarding stealth, some approaches have been addressed in this review, such as the use of zwitterionic proteins or those that have a dysopsonic effect (e.g., CD47) [121], as well as the use of polymeric (e.g., PEG) [14] and biomimetic coatings (e.g., erythrocyte, macrophage, and cancer membranes) [133,136,140].

Understanding how NPs interact with body fluids is therefore critical to their application. Since the composition of the corona varies from patient to patient, the concept of “personalized protein corona” [141] and “disease-specific corona” could be part of personalized/targeted medicine. The changes in PC could also be useful as early biomarkers for many diseases [142] and have attracted interest in the scientific community. Comparison of HP from healthy individuals with those from individuals affected by various diseases (e.g., breast cancer, diabetes, rheumatism, hypercholesterolemia, hemophilia A and B, thalassemia, common cold) has shown that changes in protein composition can affect the PC of hydrophobic sulfonate PS-NPs and hydrophilic amorphous SiO_2_ NPs [143]. In the context of a disease-specific PC, the patient’s PC profile could be used as a detection tool for difficult-to-detect biomarkers. In this sense, the PC of AuNPs exposed to the serum of breast cancer and healthy patients was analyzed to identify cancer-associated inflammatory markers. The study revealed that AuNPs exposed to breast cancer sera contained neutrophil-derived granule proteins (i.e., properdin, myeloperoxidase (MPO) and MM-9) that were helpful in distinguishing different breast cancer subtypes, as well as healthy individuals [144]. In addition, ovarian and liver cancer proteins obtained from patient lysates were used to coat AuNP and SiO_2_ NPs. The study showed that only coated AuNP promoted the proliferation of T lymphocyte and maturation of DCs without causing cytotoxicity, while coated-SiO_2_ did not [145]. This is an encouraging result for the use of PC on the surface of NPs to create a simple and customizable NPs-based nanocarrier for cancer vaccine applications. However, further studies on this topic are needed.

**Figure 9:**
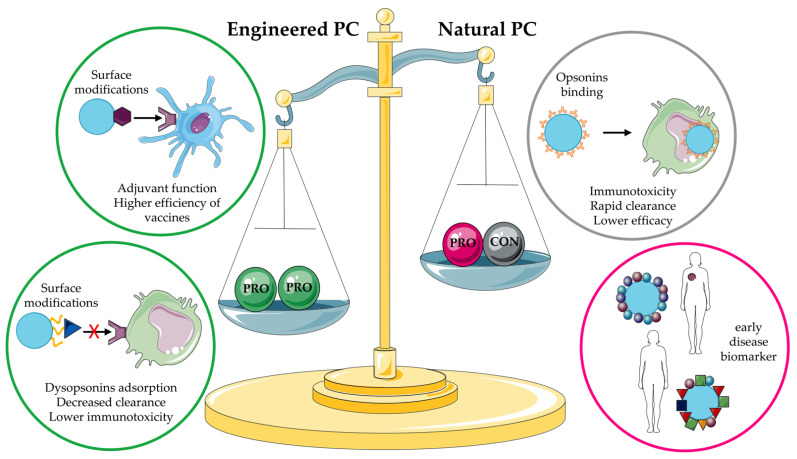
Pros and cons of the natural and engineered PCs. The figure was partly generated using Servier Medical Art by Servier, licensed under the Creative Commons Attribution 3.0 Unported Licence. PC: protein corona.

In summary, the PC is a key player in the fate of NPs in vivo and can be used to customize engineered properties and achieve better results in terms of in vivo stealth, targeting, early disease diagnostics, and personalized medicine (Figure 9). Combining PC characterization methods such as proteomics and bioinformatics could uncover important information about disease development and new therapeutic targets.

## Figures and Tables

**Table 1:** Dysopsonins.

Name of the Protein	How Dysopsonins Affect Uptake of NPs in Macrophages	Type of NP Studied
HRG	Stealth properties	SiO_2_-NPs [51,52]
Apos	Modulation of C activation	PEG-NPs (Apo-J) [14]
		PS-NPs [14]
		SiO2-NPs (Apo-J) [74]
		AgNPs (Apo-J) [74]
	Decrease in clearance speed	Liposomes (Apo-AI) [75]
HSA	Stealth properties	PS microparticles [76]
		HA-NPs [77]
	Decrease in clearance speed	PHBHHx NPs (BSA) [74]
SP-A and SP-D	Hypothesis: inhibition of NPs’ agglomeration	PS-NPs (SP-A) [23]
		CNTs (SP-D) [23]

HRG: histidine-rich glycoprotein; SiO_2_-NPs: silica nanoparticles; AgNPs: silver NPs; Apos: apolipoproteins; HSA: human serum albumin; PS: polystyrene; HA: hyaluronic acid; BSA: bovine serum albumin; PHBHHx: poly-3-hydroxybutyrate-co-3-hydroxyhexanoate; SP-A and SP-D: surfactant protein A and D; CNTs: carbon nanotubes.

**Table 2:** PC and interaction with immune system cells other than macrophages.

Cell Type	Type of NP Studied	Cell’s Receptors Involved	Effect
Dendritic cells	C-opsonized FeO-DEX NPs [112]	CR3	Increased NPs’ binding/uptake Lower MCHII and CD86 expression Lower responsiveness to stimuli
	OVA-loaded LPS-modified (PLGA)-NPs [113]	TLRs and NLRs	Increased uptake Stimulation of CD8+ T cell responses
Neutrophils	DEX-coated NPs BNF-starch NPs [112]	CR3 or SR-A	Increased clearance
	IgG-coated PEG-SWCNTs [114,115,116]	IgG mediated Interaction	Neutrophil activation ROS and MPO release
B cells	C-opsonized FeO-DEX NPs [112]	CR-1/2	Decrease in CD86 Lower responsiveness to stimuli
T cells	Unfolded proteins-coated CNTs [106]	Not addressed	Increase in effector T helper cells Reduction of naïve T cells

C: complement; FeO: iron oxide; DEX: dextran; NPs: nanoparticles; CR: complement receptor; MCHII: major histocompatibility complex II; OVA: ovalbumin; LPS: lipopolysaccharide; PLGA: poly(lactic-co-glycolic acid); TLRs: Toll-like receptors; NLRs: nucleotide oligomerization domain (NOD)-like receptors; BNF: bionized nanoferrite; SR: scavenger receptor; PEG: polyethylene glycol; SWCNTs: single walled carbon nanotubules; IgG: immunoglobulin G; ROS: reactive oxygen species; MPO: myeloperoxidase.

## Data Availability

Not applicable.
